# Two new phragmotic ant species from Africa: morphology and next-generation sequencing solve a caste association problem in the genus *Carebara* Westwood

**DOI:** 10.3897/zookeys.525.6057

**Published:** 2015-10-05

**Authors:** Georg Fischer, Frank Azorsa, Francisco Hita Garcia, Alexander S. Mikheyev, Evan P. Economo

**Affiliations:** 1 Okinawa Institute of Science and Technology Graduate University, 1919–1 Tancha, Onna-son 904–0495, Japan; 2 División de Entomologia, Centro de Ecologia y Biodiversidad, CEBIO. Lima, PERU; 3 Department of Natural History – Zoology, Hessisches Landesmuseum Darmstadt, Friedensplatz 1, 64283 Darmstadt, Germany; 4 Department of Ecology and Evolutionary Biology, Museum of Zoology, University of Michigan, 830 N University Street, Ann Arbor, MI 48701, USA

**Keywords:** Phragmosis, new species descriptions, Hymenoptera, Formicidae, *Carebara lilith*, *Carebara phragmotica*, worker polymorphism, RAD-seq, Afrotropics, Kenya, Ivory Coast

## Abstract

Phragmotic or “door head” ants have evolved independently in several ant genera across the world, but in Africa only one case has been documented until now. *Carebara elmenteitae* (Patrizi) is known from only a single phragmotic major worker collected from sifted leaf-litter near Lake Elmenteita in Kenya, but here the worker castes of two species collected from Kakamega Forest, a small rainforest in Western Kenya, are studied. Phragmotic major workers were previously identified as *Carebara elmenteitae* and non-phragmotic major and minor workers were assigned to *Carebara thoracica* (Weber). Using evidence of both morphological and next-generation sequencing analysis, it is shown that phragmotic and non-phragmotic workers of the two different species are actually the same and that neither name – *Carebara elmenteitae* or *Carebara thoracica* – correctly applies to them. Instead, this and another closey related species from Ivory Coast are both morphologically different from *Carebara elmenteitae*, and thus they are described as the new species *Carebara phragmotica* **sp. n.** and *Carebara lilith* **sp. n.**

## Introduction

The ant genus *Carebara* is highly diverse with about 250 named taxa to date (Bolton 2014), while the true diversity is probably much higher due to a large number of undescribed species (Fischer et al. 2014). For the vast majority of this diversity virtually nothing is known about their respective ecologies, and data about species’ biogeographic distributions is still incomplete. Apart from the conspicuous, mass-raiding marauder ants of the former genus *Pheidologeton* (now *Carebara*, see Fischer et al. 2014), most of the species are minute in size, often with very cryptic lifestyles, making field observation difficult.

Due to a lack of comprehensive revisions and identification keys for Old World *Carebara*, identifications are challenging. On a regional level, however, taxonomic treatments exist for the Arabian Peninsula (Sharaf and Aldawood 2013), Taiwan (Terayama, Lin and Eguchi 2012), India (Bharti and Kumar 2013), and Fischer et al. (2014) revised the newly defined and mostly Afrotropical *Carebara polita* group. Weber’s (1950) revision for the Afrotropical *Oligomyrmex* species is outdated and, as it does not contain a key, is also of very limited use for identifications of the treated species. For the New World, Fernández (2004) published a valuable revision of *Carebara* with a provisional key, where he synonymized the former genera *Oligomyrmex* (Mayr), *Paedalgus* (Forel), and *Afroxyidris* (Belshaw & Bolton) with *Carebara* and defined species complexes based on worker morphology. As several previous studies showed (e.g. Ettershank 1966, Fernández 2004, Fischer et al. 2014, and Azorsa and Fisher in revision) all of the synonymized genera were morphologically poorly delimited from *Carebara* and thus treated as polyphyletic units. Bharti and Kumar (2013) also pointed out the necessity to restructure Fernández’ New World species group definitions in order to incorporate the much more species-rich but poorly studied Old World fauna. Undersampling in many tropical and sub-tropical areas and especially in non-epigaeic strata is still a major issue for *Carebara* taxonomy and biogeography, and contributes to major gaps in our knowledge. Hence, more ecological and taxonomic studies in these areas are needed in order to better understand the evolution and biology of this interesting and diverse genus.

Taxonomic research in ants heavily depends on dry specimens in entomology collections and associated collection-based data, but field observations can contribute valuable insights, with the potential to improve species boundaries. The main obstacle from ecological surveys using standardized, passive collection methods (e.g. leaf-litter extraction and pitfall trapping), are disassociated specimens from different castes or subcastes. Especially in genera with distinct worker di- and polymorphism, this can create problems of inflated diversity counts. As in the hyperdiverse genus *Pheidole* Westwood, workers of many *Carebara* species are divided into two distinct subcastes, minor and major workers (or soldiers), with additonal subcastes and intermediates present in several species (Azorsa and Fisher in revision, Fischer et al. 2014). While the major workers’ most important tasks are chopping and transportation of larger prey and the defence of foraging trails and the nest, the main function of phragmotic workers is blocking nest entrances against intrusion of other predatory ants and invertebrates (Hölldobler and Wilson 1990).

Phragmosis in ants (truncated body parts – usually the head – used for plugging nest entrances) has evolved independently in the diverse ant genera *Camponotus* Mayr (*Colobopsis*, *Hypercolobopsis*), *Cephalotes* Latreille, *Colobostruma* Wheeler (*Carebara leae*), *Crematogaster* Lund (*Colobocrema*), *Pheidole* Westwood (*Pheidole colobopsis*, *Pheidole lamia*), but also in other genera, such as *Blepharidatta* Smith, (*Blepharidatta conops*), *Tetraponera* Smith (*Tetraponera phragmotica*) and *Carebara* Westwood (Brandão et al. 2001, Hölldobler and Wilson 1990). Phragmosis is most strongly developed in the New World arboreal genus *Cephalotes*, where usually all castes (queens, and major and minor workers) have highly adapted shield-like head morphologies that enable them to plug their nest entrance without exposing eyes, antennae or mandibles to any would-be intruders. Wheeler and Hölldobler (1985) discussed phragmosis in *Cephalotes* and reported the discovery of “glandular openings” on the cephalic shield that supposedly excrete fibrous material, covering the head in a dense layer of organic material and most likely serving as camouflage of the head that plugs the nest entrance. A very similar phragmotic head shape has evolved independently in some Old World species of *Carebara*, where a special major worker subcaste occurs in addition to regular major and minor workers.

**Figure 1.**
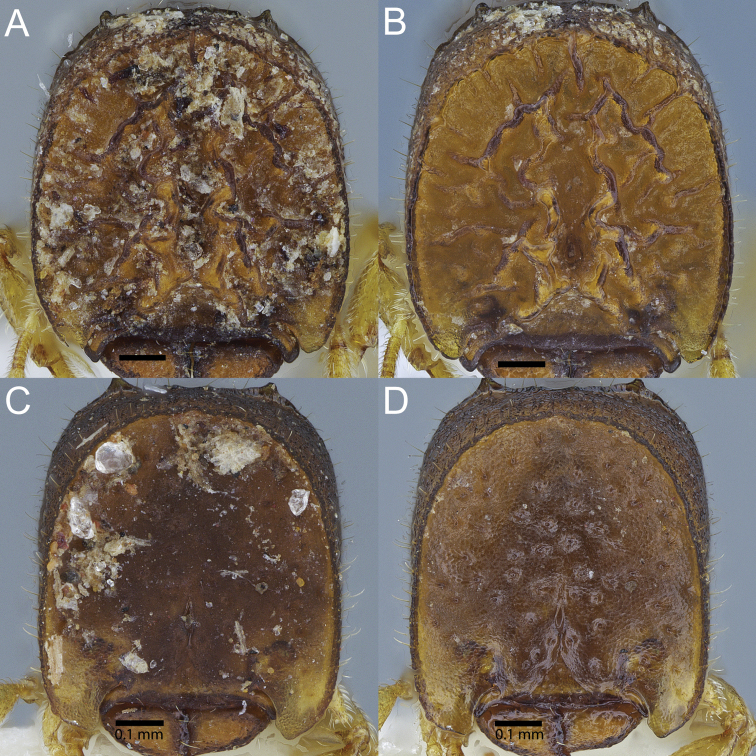
**A, B** full-face view of phragmotic major workers of *Carebara phragmotica* sp. n. and **C, D** *Carebara lilith* sp. n. **A, C** on the left side the head is depicted in the state that it was found in the samples, with debris sticking to cephalic shield **B, D** right view: same specimens with debris removed in ultrasonic bath.

Heads in the shape of a saucer or a concave shield protecting eyes, antennae and mandibles from possible injury by attackers may have evolved convergently in major workers of the Southeast Asian *Carebara butteli* (Forel), *Carebara nayana* (Sheela & Narendran) and in the subsequently treated Afrotropical *Carebara* species. The cephalic shields in the two newly described species were found to be covered by a layer of debris (soil, maybe organic material; see Fig. 1), which may serve as camouflage to make the ant blend in with the soil around the nest entrance.

During field work between 2005 and 2009 in Kakamega Forest, Western Kenya, phragmotic *Carebara* workers have been collected from seven leaf-litter samples (out of 300+), along with workers of four other species of the genus (Hita Garcia et al. 2009, Hita Garcia et al. 2013). Since most leaf-litter samples contained multiple species, their various worker subcastes were often intermixed and had to be re-associated during sorting. Because of this association problem and because of their morphological isolation due to the highly derived head shapes, the phragmotic workers were first identified as *Carebara elmenteitae* (Patrizi). The sympatrically occuring workers of the other four species were identified as *Carebara* GF4, *Carebara* GF5, *Carebara polita* (Santschi), and *Carebara thoracica* (Weber) (for updated species IDs see Table 1). At first, there was no evidence for a relationship between the phragmotic workers and those of any other species.

**Table 1.** Updated species IDs of Kakamega Forest *Carebara* specimens used in this study, compared to old IDs in Hita Garcia et al. (2009, 2013).

Updated species ID	Old species ID	Worker subcaste
*Carebara phragmotica* sp. n.	*Carebara elmenteitae*	phragmotic
*Carebara phragmotica* sp. n.	*Carebara thoracica* (10 ant. segments)	minor & major
*Carebara thoracica*	*Carebara thoracica* (9 ant. segments)	minor & major
*Carebara silvestrii*	*Carebara* GF4	minor & major
*Carebara alluaudi*	*Carebara* GF5	minor & major
*Carebara polita*	*Carebara polita*	minor & major

However, more recently we inferred that they were most likely an additional subcaste to non-phragmotic major and minor workers which were falsely identified as *Carebara thoracica*. Our assumption was based primarily on the specimens (described here as *Carebara lilith* sp. n.) found during a visit to the MHNG ant collection in Geneva during November 2013. For *Carebara lilith*, a phragmotic worker had been collected together with two minor workers. These specimens, with morphologies highly similar to the phragmotic workers and minor workers found in Kakamega Forest leaf-litter samples, were the initiator of our following investigations. Morphological key features such as antennal segmentation, petiole and postpetiole morpholgy, sculpture patterns, and pilosity were examined of all Kakamega species co-occuring with the phragmotic workers. That way it is possible to infer the relationship between specimens identified as *Carebara elmenteitae* (phragmotic workers) and *Carebara thoracica* (non-phragmotic workers with 10 antennal segments) and to exclude the other *Carebara* species with non-matching morphologies as likely conspecifics. For a more rigorous test of our hypotheses de-novo DNA sequencing (RAD-seq) and analysis of the relevant material was used. As outgroup material selected the sympatric and morphologically related, yet distinct, *Carebara alluaudi*, was selected as well as the more distantly related *Carebara sylvestrii*, and two undetermined Chinese *Carebara* morphospecies (collected by Liu et al.).

**Figure 2.**
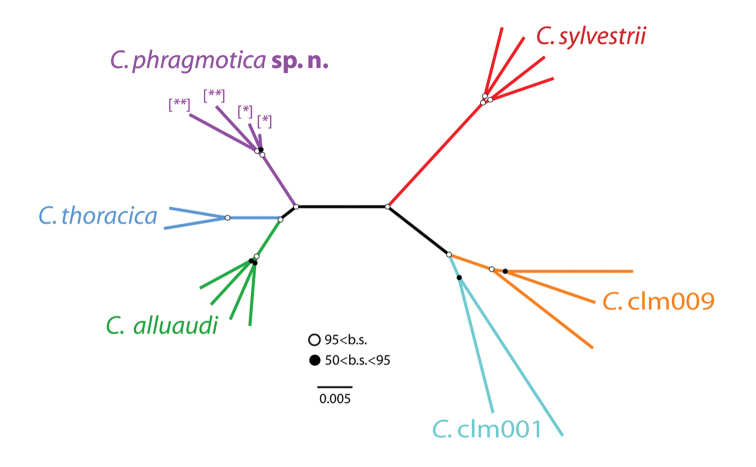
Maximum likelihood tree of sequenced *Carebara* specimens reconstructed with ExaML v3.0.14. Most nodes are supported with more than 0.95% of bootstraps (represented by open circles) except for five, which have between 0.5 and 0.95% support (black circles). The tree shows the division between *Carebara phragmotica* sp. n. specimens with ten antennal segments (previous IDs: [*] *Carebara elmenteitae*, [**] *Carebara thoracica*) and all other sampled specimens, including *Carebara thoracica* with nine antennal segments, which are closer related to 9-segmented *Carebara alluaudi*.

As a result of these morphological and genetic studies, two new phragmotic *Carebara* species, *Carebara lilith* sp. n. from Ivory Coast and *Carebara phragmotica* sp. n. from Kenya are described, that both are likely related to *Carebara elmenteitae* (Fig. 2). All three species have the phragmotic major worker subcaste and although *Carebara elmenteitae* is known from a phragmotic worker only, we assume the missing workers to be morphologically related to the workers of our newly described species. In addition to detailed descriptions and high-resolution composite images of the two new species, a species-level identification key is provided for the three phragmotic species, as well as a discussion for their taxonomic placement within the Afrotropical *Carebara* fauna.

## Material and methods

All specimens in this study were examined with a Leica MZ165x stereo microscope (up to maximum magnification of 120×), and measured with an orthogonal pair of micrometers.

Morphological characters and measurements used in this publication are the same as in Fischer et al. (2014), which are mostly derived from Bolton (1994, 2003), Bolton and Belshaw (1993), Belshaw and Bolton (1994), Ettershank (1966), Fernández (2004, 2006, 2010). For sculpture characters we refer to Harris (1979) and for different pilosity patterns we use the five inclination types described by Wilson (1955).

High-resolution images were created using Leica DFC 425 and DFC 450 cameras in combination with the Leica Application Suite software (version 3.8) and Helicon Focus 6 software. All images were individually edited using Photoshop and combined into plates with Adobe Ilustrator software. Images can be viewed and downloaded at www.AntWeb.org.

Most of the material studied in this publication is located in ZFMK in Bonn, the holotype of *Carebara phragmotica* is deposited in ZFMK, the material of *Carebara lilith* sp. n. belongs to the MHNG ant collection in Geneva. Paratypes of *Carebara phragmotica* sp. n. will be deposited in the NMK (Nairobi), in the MCZC (Cambridge, MA), and in the MHNG (Geneva).

### Abreviations of depositories

BMNH British Museum of Natural History, London, UK

DCZU University of Calicut, Department of Zoology

IEGG Istituto di Entomologia “Guido Grandi” Università di Bologna, Bologna, Italy

MHNG Muséum d’Histoire Naturelle de la Ville de Genève, Geneva, Switzerland

MCZC Museum of Comparative Zoology, Cambridge, Mass. U.S.A.

NMK The National Museums of Kenya, Nairobi

ZFMK Zoologisches Forschungsmuseum Alexander Koenig, Bonn, Germany

### DNA sequencing and analysis

Phylogenetic analysis of restriction site-associated DNA (RAD-seq) was used (Baird et al. 2008) to test the relationship of phragmotic workers previously identified as *Carebara elmenteitae* with non-phragmotic major and minor workers identified as *Carebara thoracica* and containing specimens with both, nine and ten antennal segments (results in Fig. 2). Outgroup taxa are two species from Kakamega Forest occuring in sympatry with the above mentioned ingroup taxa (*Carebara sylvestrii* and *Carebara alluaudi*) and two other *Carebara* species from Yunnan, China (*Carebara* clm001 and *Carebara* clm009). The 19 specimens included in the analysis (Table 2) comprise focal taxa plus outgroups for phylogenetic context.

**Table 2.** List of *Carebara* specimens used for DNA sequencing (* phragmotic workers, previously identified as *Carebara elmenteitae*; ** minor workers, previously identified as *Carebara thoracica* (Hita Garcia et al. 2009, Hita Garcia et al. 2013).

Species ID [number of antennal segments]	Specimen code	Basepairs analysed	DDBJ experiment ID	Country
*Carebara phragmotica* sp. n.* [10]	CASENT0738556	333886	DRX032389	Kenya
*Carebara phragmotica* sp. n.* [10]	CASENT0738559	948331	DRX032390	Kenya
*Carebara phragmotica* sp. n.** [10]	CASENT0738560	783551	DRX032399	Kenya
*Carebara phragmotica* sp. n.** [10]	CASENT0738561	621109	DRX032400	Kenya
*Carebara thoracica* [9]	CASENT0738564	454125	DRX032401	Kenya
*Carebara thoracica* [9]	CASENT0738565	456134	DRX032402	Kenya
*Carebara alluaudi* [9]	CASENT0738554	780239	DRX032395	Kenya
*Carebara alluaudi* [9]	CASENT0738555	665163	DRX032396	Kenya
*Carebara alluaudi* [9]	CASENT0738566	880370	DRX032397	Kenya
*Carebara alluaudi* [9]	CASENT0738567	884559	DRX032398	Kenya
*Carebara silvestrii* [11]	CASENT0738557	303910	DRX032391	Kenya
*Carebara silvestrii* [11]	CASENT0738558	414766	DRX032392	Kenya
*Carebara silvestrii* [11]	CASENT0738562	525888	DRX032393	Kenya
*Carebara silvestrii* [11]	CASENT0738563	428646	DRX032394	Kenya
*Carebara* clm001 [9]	CASENT0735929	155549	DRX032384	China
*Carebara* clm001 [9]	CASENT0735930	26192	DRX032385	China
*Carebara* clm009 [9]	CASENT0735913	220323	DRX032386	China
*Carebara* clm009 [9]	CASENT0735914	78455	DRX032387	China
*Carebara* clm009 [9]	CASENT0735915	70339	DRX032388	China

DNA was non-destructively extracted from each specimen following Tin et al. (2014) by soaking it overnight in a chaotropic buffer. The DNA was then bound to magnetic beads and washed prior to library preparation. RAD-tag libraries were then prepared as in Tin et al. (2015) using a Biomek® FXP Laboratory Automation Workstation (Beckman Coulter) to perform all of the liquid handling steps up to PCR. Sequencing was performed on an Illumina Hi-Seq platform. The barcodes were designed following Bystrykh (2012). Trimmomatic (Bolger et al. 2014) was used to filter by quality and trim the sequences to 55bp (parameters SLIDINGWINDOW:8:10 MINLEN:41 CROP:41). The FASTq files containing DNA sequence reads were uploaded to the DNA Data Bank of Japan (DDBJ, http://www.ddbj.nig.ac.jp/, bioproject_id: PRJDB3919). We used PyRAD v3.0.4 (Eaton 2014) for *de novo* assembly of RAD loci (parameters: *Mindepth=6, NQual=5, Wclust=0.88, MinCov=4, MaxSH=3*, otherwise default) and performed a maximum likelihood (ML) phylogenetic analysis on the full alignment (1.78 million bp) with ExaML v3.0.14 (Kozlov et al. 2015). The GTR+G nucleotide substitution model was chosen, as it was the only option implemented in ExaML suitable for a dataset of this size. To evaluate support for the ML topology, 1000 bootstraps were performed in a combination of ExaML and RaxML v8.0.0 (Stamatakis 2014), following the procedure described in the ExaML manual. The alignment, inferred topology, and further details on the procedure for the ML search are available on datadryad.org (http://datadryad.org/review?doi=doi:10.5061/dryad.1jc33).

### Measurements and indices

The following measurements are illustrated in Figure 1 in Fischer et al. (2014):

HL
*head length*: maximum distance from midpoint of anterior clypeal margin to midpoint of posterior margin of head, measured in full-face view; in majors, measured from midpoint of tangent between anterior-most position of clypeus to midpoint of tangent between posterior-most projection of the vertex.

HW
*head width*: measured at widest point of head, in full-face view behind eye level.

SL
*scape length*: maximum scape length, excluding basal condyle and neck.

EL
*eye length*: maximum diameter of compound eye measured in oblique lateral view.

MFL
*metafemur length*: measured from junction with trochanter to junction with tibia.

MTL
*metatibia length*: measured from junction with femur to junction with first tarsal segment.

MDL
*mandible length*: maximum length, measured in oblique frontolateral view, from apex to lateral base.

PNW
*pronotal width*: maximum width of pronotum measured in dorsal view.

WL
*Weber’s length*: diagonal length of mesosoma in profile from anterior point of pronotal slope and excluding neck, to posteroventral margin of propodeum.

PSL
*propodeal spine length*: in dorsocaudal view, with apex of measured spine, its base, and center of propodeal concavity between both spines in focus: measurement is taken from apex to base along one axis of a dual-axis micrometer, which is aligned along length of spine, while second axis crosses base of measured spine, and connects base with center of propodeal concavity.

PTL
*petiole length*: maximum diagonal length of petiole, measured in profile, from most anteroventral point of peduncle, at or below propodeal lobe, to most posterodorsal point at junction to first helcial tergite.

PTH
*petiole node height*: maximum height of petiolar node measured in lateral view from highest (median) point of node, orthogonally to ventral outline of node.

PTW
*petiole node width*: maximum petiolar node width, measured in dorsal view.

PPL
*postpetiole length*: maximum length of postpetiole, measured in profile, from anterior beginning of dorsal slope to posterior juncture of postpetiole and second helcial tergite.

PPH
*postpetiole height*: maximum height of postpetiole, measured in profile, from the highest (median) point of node to lowest point of ventral face, often in an oblique line.

PPW
*postpetiole width*: maximum width of postpetiole, measured in dorsal view.

### Indices

CI
*cephalic index*: HW / HL × 100

SI
*scape index*: SL / HW × 100

MDI
*mandible index*: MDL / HW × 100

EI
*eye index*: EL / HW × 100

FI
*metafemur index*: MFL / HW × 100

PSLI
*propodeal spine index*: PSL / HW × 100

LPpI
*lateral postpetiole index*: PPL / PPH × 100

DPpI
*dorsal postpetiole index*: PPW / PPL × 100

PpWI
*postpetiole width index*: PPW / PTW × 100

PpLI
*postpetiole length index*: PPL / PTL × 100

PpHI
*postpetiole height index*: PPH / PTH × 100

## Results

### Species synopsis

*Carebara elmenteitae* (Patrizi)

*Carebara lilith* Fischer, Azorsa & Hita Garcia, sp. n.

*Carebara phragmotica* Fischer, Azorsa & Hita Garcia, sp. n.

### Other *Carebara* species with phragmotic workers

Pheidologeton (Lecanomyrma) butteli Forel, 1913: 56, fig. S (s.w.) SRI LANKA, Peradeniya, Experiment Station (*v. Buttel*) (MHNG) [examined]. Combination in *Aneleus*: Emery 1924: 215; in *Oligomyrmex*: Ettershank 1966: 123; in *Carebara*: Fernández 2004: 235.

*Neoblepharidatta nayana* Sheela & Narendran, 1997: 89, figs 1-4 (s., not q. as stated) INDIA, Kerala, Iritty Forest near Aaralam farm, 16.xii.1995 (*Sheela*) (DCZU) [not examined]. Combination in *Oligomyrmex*: Bolton 2003: 273. Combination in *Carebara*: Fernández 2004: 196 (by implication).

### Preliminary definition of the *Carebara phragmotica* clade

The three species treated are loosely defined here as a clade based on the presence of and morphological similarity between the phragmotic workers. We do not claim that these species form a monophyletic or exclusive clade within the genus *Carebara*. Although we think that a sister-species relationship between them is the most likely hyphothesis, it is nevertheless possible that morphological similarities are due to convergence and that they are not closely related. Another hypothesis is that they are indeed very closely related, but forming a monophyletic group with other species that do not possess phragmotic workers. As highly visible in the systematic history of *Carebara* and its constituent species and synonymous genera, the definition of species groups or even genera based on morphology alone can be both, a tedious and sometimes frustrating approach with taxonomic group definitions changing frequently (see Ettershank 1966, Fernández 2004, Fischer et al. 2014).

Since the emergence of increasingly affordable DNA-sequencing methods generating more comprehensive data-output as compared to Sanger sequencing (e.g. genome and next-generation sequencing), higher emphasis should be placed on combined taxonomic and genetic analyses in order to reduce discrepancies between both approaches. For the *phragmotica* clade and the majority of *Carebara* species, only a large-scale taxonomic treatment and/or a near-comprehensive phylogenetic analysis of the whole genus would be able to provide the level of confidence needed for definition of exclusive and monophyletic species groups. However, future studies are necessary to close these gaps in our taxonomic understanding of the genus *Carebara* Westwood.

### Shared characters of *Carebara phragmotica* clade species (all worker subcastes)

(the characters listed below may not be autaphomorphic, since the majority of Afrotropical taxa remain poorly characterized and because of possible convergent evolution)

Phragmotic major workers present, with oval cephalic shield and anterolateral lobes covering the lateral base of the mandible. Antennae with 10 segments and 2-segmented club, the apical segment between combined length of antennal segments 3 to 9 and length of remainder of funiculus (antennal segments 2-9). Antennal scape relatively short, in minor workers failing to reach the posterior head margin by about lenght of 9th antennal segment, in majors of *Carebara phragmotica* ending at about midlength of head (SI 46-49), in phragmotic workers distinctly shorter and reduced (SI 21-34). Mandibles triangular and masticatory margin with five teeth, mandibles of phragmotic workers reduced and very small, about half as long as those of major workers in *Carebara phragmotica* (MDI 24-28). Anterior margin of clypeus in phragmotic workers very wide and straight to medially concave. Eyes minute and consisting of one ocellus, in phragmotic workers reduced and almost invisible, single median ocellus often present in major workers of *Carebara phragmotica*, but invisible or absent in phragmotic workers. Metanotal groove in profile impressed and propodeum higher than long. Propodeal teeth developed, relatively small and apically rounded to short-triangular and acute. Petiole quite massive in profile, with moderately long peduncle, a small, anteriorly pointing anteroventral tooth, and often with conspicuously convex ventral bulge, in dorsal view almost as wide as (minor workers) to wider than propodeal dorsum (majors and phragmotic workers). Postpetiole roundly subrectangular in dorsal view, between 1.2 and 1.5 times wider than petiole. In minor workers (of *Carebara phragmotica* and *Carebara lilith*) sculpture absent from head, promesonotum, dorsum of postpetiole and gaster.

### Delimitation from other *Carebara* groups and species in the Afrotropical region

Here a general account of the Afrotropical *Carebara* fauna is given as well as information on how to differentiate species belonging to the *phragmotica* clade from the remainder of *Carebara* species that were found and described for the region, not including Madagascar. They can be devided into several groups of morphologically related species, some of which correspond to the preliminary groups defined by Fernández (2004): *lignata* complex for *Carebara sensu stricto* (before synonymization of *Oligomyrmex* Mayr), *escherischi* complex for former *Paedalgus* species, and with the former *Oligomyrmex* species roughly corresponding to the *concinna* complex, although it was defined for New World species, which have eleven antennal segments, contrasting to mostly 9- and 10-segmented Old World species. Two of these New World *concinna* complex species, *Carebara brevipilosa* Fernández and *Carebara urichi* (Wheeler), are now included in the *polita* group, but exact phylogentic relationships within and between the different faunas are still unresolved. Because we want to avoid creating polyphyletic species groups, we leave the definition of systematic species groups to larger-scale studies in the future.

Afrotropical *Carebara* species belonging to the former genus *Pheidologeton* are: *Carebara aberrans* (Santschi) (queen), *Carebara diversa standfussi* (Forel), *Carebara hammoniae* (Stitz), *Carebara hostilis* (Smith), *Carebara kunensis* (Ettershank), *Carebara mayri* (Forel), *Carebara solitaria* (Stitz) (queen), and *Carebara volsatella* (Santschi) (male). They are mainly characterized by possessing eleven antennal segments, a markedly polymorphic worker caste with several intermediate worker subcastes, comparatively large, multi-facetted eyes, minor workers with antennal scapes usually surpassing the posterior head margin, and large major workers, usually with one to several large occeli present. Morphologically, this group is closest to some species of the *polita* group, e.g. *Carebara nicotiana* (Arnold) and *Carebara polita* (Santschi). The *polita* group can be distinguished from other *Carebara* by antennae with eleven segments (but only nine in *Carebara madibai* Fischer & Azorsa), eyes reduced, in minor workers usually consisting of a single ocellus, in majors sometimes larger and multi-facetted, but smaller than in former *Pheidologeton* species, major workers usually with high, weakly squamiform petiole node, and minor workers with postpetiole significantly longer than high in profile (Fischer et al. 2014). In Africa the *polita* group includes: *Carebara madibai* Fischer & Azorsa, *Carebara perpusilla* (Emery), *Carebara polita* (Santschi), *Carebara nicotianae* Arnold, *Carebara silvestrii* (Santschi), and *Carebara villiersi* (Bernard). Species described in or assigned to the former genus *Pheidologeton* (Forel) (= *escherischi* complex in Fernández 2004) are: *Carebara distincta* (Bolton & Belshaw), *Carebara octata* (Bolton & Belshaw), *Carebara pisinna* (Bolton & Belshaw), *Carebara rara* (Bolton & Belshaw), *Carebara robertsoni* (Bolton & Belshaw), *Carebara sarita* (Bolton & Belshaw), *Carebara sudanensis* (Weber) (queen), and *Carebara termitolestes* (Wheeler). They all share morphological characters that distinguish them from other *Carebara* species, i.e. nine antennal segments, mandibles with four teeth, metanotal groove not impressed, propodeum oblique in profile and declining towards posterior end without distinct angle, and propodeal teeth absent. Species of *Carebara sensu stricto* (definition before Fernández 2004, = *lignata* complex) are characterized by small workers and usually much larger queens, the workers usually with nine antennal segments, mandibles with three to four teeth, eyes and propodeal teeth absent, the propodeal dorsum often rounding into the posterior declivity without any angle. Species with matching morphologies are *Carebara arnoldi* (Forel), *Carebara guineana* Fernández, *Carebara junodi* Forel, *Carebara osborni* Wheeler, *Carebara vidua* Smith, Carebara vidua var. fur Santschi, *Carebara wheeleri* Ettershank (replacement name for *Carebara silvestrii* Santschi). *Carebara ampla* Santschi and its subspecies, *Carebara bartrumi* Weber, *Carebara langi* Wheeler, *Carebara sicheli* Mayr, and *Carebara sudanica* Santschi are all known from queens and/or males only, but their queens are usually very large and are morphologically close to *Carebara vidua*. It is unclear, however, how many species described by alates are synonymous with worker-based species. This has to be investigated in future studies, but colony collections with associated workers and alates are rare and difficult to achieve in a systematic way. Workers of *Carebara fayrouzae* Sharaf, which occurs in Saudi Arabia, also have nine antennal segments and minor worker and queen morphologies closely match those of the above listed species in *Carebara s. str.* If they they should turn out to be closely related, then *Carebara fayrouzae* would be the first species in this group of which major workers have been found and described. In that case, it would not be unlikely that other species of *Carebara s. str.* and former *Paedalgus* are not monomorphic, but di- or even polymorphic as well. As Fernández (2004) pointed out, the currently available material is insufficient to answer this question. Workers of *Carebara crigensis* (Belshaw & Bolton) – described originally in its own genus *Afroxyidris* – which are morphologically similar to *Carebara s. str.*, are characterized by antennae with ten segments, mandibles with two apical teeth plus one small basal tooth, eyes absent, and propodeum unarmed and rounded posteriorly.

Species from the *phragmotica* clade are part of a larger group of morphologically related species, which includes many taxa belonging to the former genus *Oligomyrmex* (Mayr). Before its synonymisation under *Carebara* by Fernández (2004), *Oligomyrmex* was defined by possessing nine to eleven antennal segments (rarely eight), a markedly dimorphic worker caste, a well-developed metasternal process, anterior subpetiolar process present and radial cell of wing closed (Ettershank 1966). Including the two newly described species, workers of 30 valid species and subspecies of Afrotropical *Carebara* match this character combination. Twelve of them have nine antennal segments: *Carebara alluaudi* (Santschi), Carebara alluaudi var. cataractae (Santschi), *Carebara angolensis* (Santschi), Carebara angolensis r. congolensis (Forel), *Carebara convexa* (Weber), *Carebara donisthorpei* (Weber), *Carebara frontalis* (Weber), *Carebara jeanneli* (Santschi), *Carebara latro* (Santschi), *Carebara pumilia* (Fischer, Azorsa, & Fisher; replacement name for *Carebara nana* (Santschi)), *Carebara santschii* (Weber), and *Carebara thoracica* (Weber). It has to be noted though, that Weber (1952) later found specimens of *Carebara thoracica* with both, nine and ten antennal segments, as well as specimens that had nine segments on one antenna and ten on the other one. This character polymorphism seems to be not uncommon and can be observed in a few other species as well, calling to attention the relatively high plasticidy in some of the characters that are usually used for taxonomic delimitation.

One species, *Carebara diabola* (Santschi), which was originally described in the genus *Aneleus*, has eleven antennal segments. Workers of the remaining 17 species have ten antennal segments, including *Carebara elmenteitae*, *Carebara lilith* sp. n. and *Carebara phragmotica* sp. n. All of the latter three species are probably polymorphic, with the highly derived phragmotic majors as a distinct third subcaste, but the other two worker subcastes share many morphological characters with the other 14 species of this group. Some of them are easily distinguishable from the *phragmotica* clade, but several are strinkingly similar in their outer morphologies and only a complete taxonomic treatment will be able to draw more definitive species boundaries. In the following paragraph is a short account of possibly related taxa, listing some supposedly stable characters that may be useful for their identification and delimitation.

Major workers of *Carebara acuta* (Weber) are characterized by reticulate-punctate sculpture on head dorsum, with striae anteriorly, and propodeal teeth long and acute, minor workers without visible sculpture except for striae on anterior head (Weber 1952). The majors of *Carebara africana* (Forel) (minors not described) without longitudinal rugulae on head, mandibles with six teeth, and propodeal teeth absent or reduced. *Carebara arabica* (Collingwood & Van Harten) is morphologically very similar to *phragmotica* clade specimens, but its major workers are characterized by oblique posterior head corners with a distinct angle towards the median emargination, moderately large horns on the distal part of the posterior margin, and longitudinal rugulae moderately abundant, evenly spaced; the minor workers with reduced sculpture on meso- and metapleurae and absent or reduced propodeal teeth. Major and minor workers of *Carebara arnoldiella* (Santschi) are also lacking distinct propodeal teeth and head sculpture in major workers is strongly reduced and consists of only a few weakly developed rugulae. Head shape and sculpture of *Carebara debilis* (Santschi) major workers is very similar to *Carebara phragmotica* majors, but the type specimen possesses a very large median occelus, minute, rounded propodeal teeth, petiole longer than high and petiole node in profile widely convex, postpetiole in dorsal view very broadly elliptical, and some majors with only nine antennal segments; minor workers with very reduced propodeal teeth and only nine antennal segments. *Carebara erythraea* (Emery) major worker’s head with very shallow posterior emargination, relatively few, short, longitudinal rugulae, frons and posterior sides almost smooth, propodeal teeth in major and minor workers not defined, but posterolateral lamella present. Major workers of *Carebara incerta* (Arnold), *Carebara khamiensis* (Arnold), and *Carebara lucida* (Santschi) are not described; their minors are characterized by absent or reduced propodeal teeth, short, in profile subtriangulate petiole with very short peduncle, mandibles in *Carebara incerta* and *Carebara khamiensis* with only four teeth, but five in *Carebara lucida*. The holotype of *Carebara petulca* (Wheeler), of which only the major worker is described, is characterized by densely rugulose head sculpture, a large median ocellus, small horns on the posterior head margin, relatively large eyes with six ommatidia, a distinct scutellum, and a high, posteriorly bluntly angled propodeum with two distinct teeth. Major workers of *Carebara semilaevis* (Mayr), described in its junior synonym *Carebara hewitti* (Santschi), are characterized by head densely rugulose, except for smooth anteromedian spot on frons, posterior head margin with very shallow emargination, horns absent, propodeal teeth in major and minor absent or reduced to rounded angles; minor workers petiole very short-pedunculate and about as high as long in profile. *Carebara traeghordi* (Santschi), *Carebara ugandana* (Santschi), and *Carebara vorax* (Santschi) are described only from minor workers, the former two are defined by head and promesonotal dorsum mostly smooth and shiny, propodeal teeth absent or reduced to blunt angles, the petiole in *Carebara traeghordi* being more compact than in *Carebara ugandana*, with shorter peduncle, and petiole node in profile more broadly convex and petiole ventrally convex. The head and body of *Carebara vorax* minors are covered with punctate-reticulate sculpture except for smooth spot anteromedially on frons and the anterior of pronotum, propodeum with well-developed lamella and teeth at its posterior corners, petiole relatively long-pedunculate.

## Results from DNA sequencing

**Figure 3.**
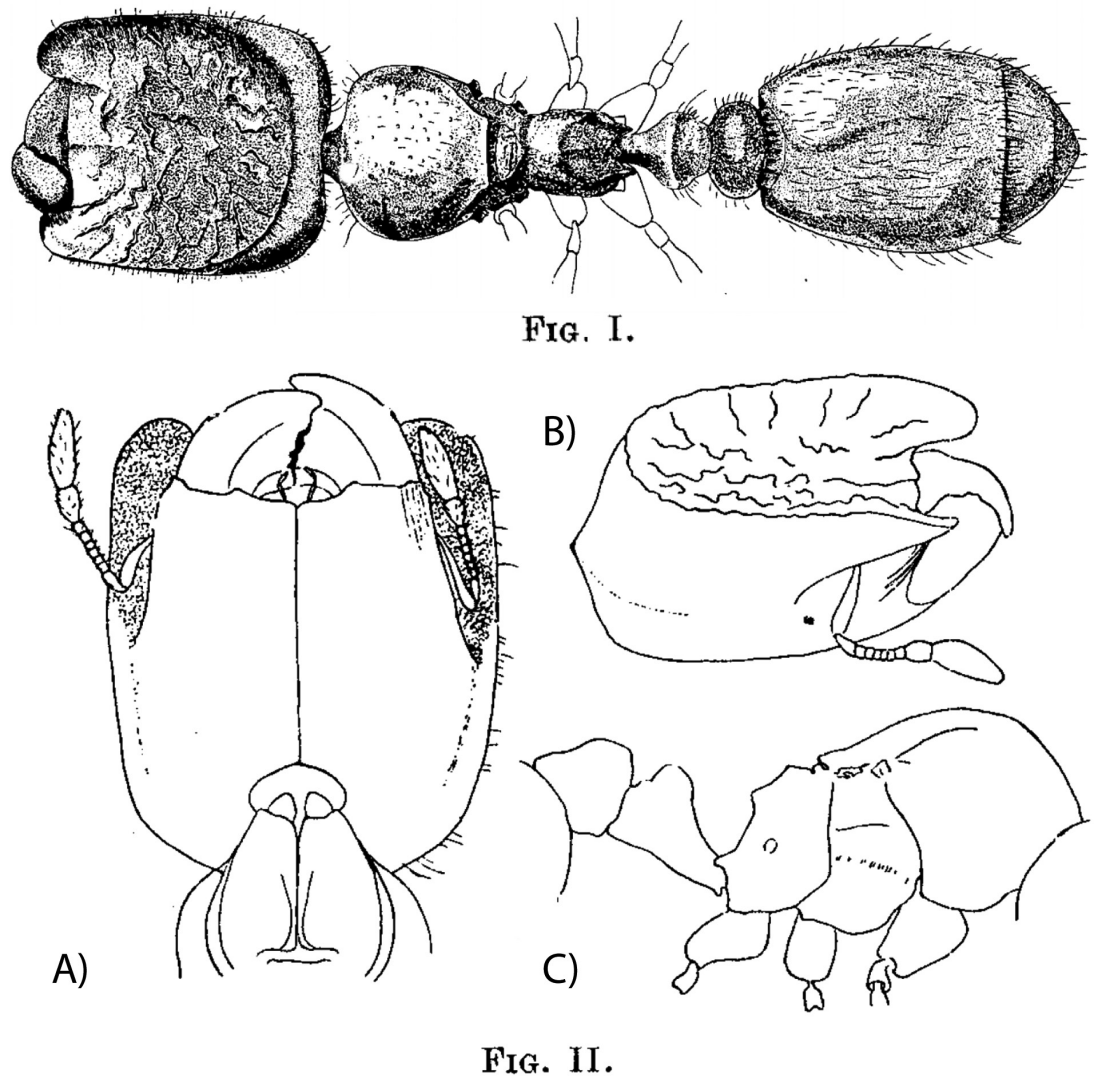
*Carebara elmenteitae* (Patrizi, 1948) – original drawings. Holotype phragmotic worker (**I**), dorsal view (**II)**: **A** head in ventral view **B** head in oblique dorsolateral view **C** mesosoma and waist in profile.

**Figure 4.**
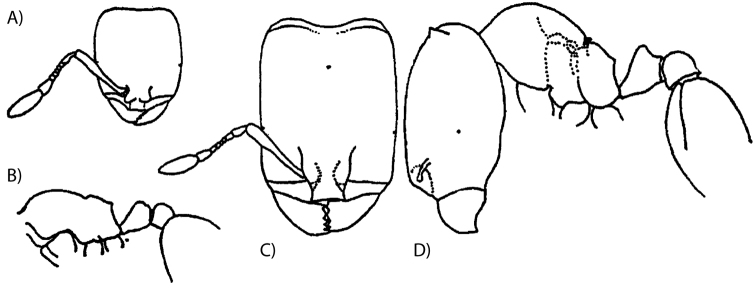
*Carebara thoracica* (Weber, 1950) – original drawings. **A** minor worker full-face view **B** minor worker profile of body **C** major worker full-face view **D** major worker profile.

Phylogenetic analysis with the RAD-seq data produced a resolved, highly supported topology (Figure 2). The phragmotic workers previously identified as *Carebara elmenteitae* (in Hita Garcia et al. 2009, Hita Garcia et al. 2013; type drawings in Fig. 3) are in a species clade together with minor worker specimens previously identified as *Carebara thoracica* (type drawings in Fig. 4). Both have ten antennal segments and belong to the new species *Carebara phragmotica*. In a separate clade, *Carebara thoracica* specimens with nine antennal segments cluster together with *Carebara alluaudi*, which also possesses 9-segmented antennae. The African *Carebara silvestrii* and the Chinese *Carebara* taxa are more distantly related (which is also reflected in different morphologies).

### Notes on phragmotic *Carebara* species

Contradicting the description of *Carebara elmenteitae* as a dealate female (queen) by Patrizi (1948), we agree with Bolton (1995) in his opinion that the described specimen is actually a major worker. As in all other examined phragmotic major workers of this group, ocelli are absent (or vestigial) and the mesosoma is clearly not built for flight, as the sclerites necessary for flying are too small and also they are fused. The drawing of the type specimen shows “scars” where in an alate queen wings would have been attached. Yet, these scars are most likely vestigial and they can be observed in the majors of many other *Carebara* species, including the two species that we describe here. However, it is possible that those specimens are ergatoid queens and that winged queens are either non-existent in this group or haven’t yet been collected or associated. In favor of this explanation could be the observation from the collection of *Carebara butteli* Forel from Sri Lanka, another, possibly convergently evolved, species with a phragmotic head. The collector, Prof. von Buttel-Reepen, noted that he found eggs, larvae, minor and major workers but no queen in the walnut-sized nest inside a termite mound (Forel 1913). The *Carebara butteli* type specimen does have a much enlarged gaster (see at AntWeb.org: CASENT0908888), suggesting that it is either physogastric, or that the majors are functioning as repletes. More collections and direct observations will be necessary, however, to draw solid conclusions. In order to learn more about species’ caste and worker evolution and their respective behaviours and functions within the colony, future field and laboratory studies ought to make an effort and investigate the ecology of these and other cryptic ant species more closely.

### Identification key for species of the *Carebara phragmotica* species clade

**Phragmotic major workers**:

**Table:** 

1	Head with distinct horns at posterior margin, cypeal margin with anterolateral lobes partly hidden under cephalic shield. Center of cephalic shield either with two highly raised, subparallel ridges, or flat with punctures and cone-shaped, gland-like structures (Figs 5A, 6A)	**2**
–	Horns on posterior border of head and anterolateral lobes of clypeal margin lacking or invisible in full-face view. Sculpture in center of cephalic shield irregularly rugose, neither flat nor with two raised ridges (Fig. 3I, II)	***Carebara elmenteitae*** (Patrizi) (Kenya) [major & minor workers unknown].
2	Cephalic shield lobes longer than and covering most of anterolateral lobes of clypeus. Center of cephalic shield flat, punctate and with cone-shaped, gland-like structures (Fig. 5A)	***Carebara lilith* sp. n.** (Ivory Coast) [major workers unknown]
–	Anterolateral lobes of clypeus longer than those of cephalic shield and anteriorly surpassing them. Center of cephalic shield with two highly raised, subparallel ridges (Fig. 6A,B)	***Carebara phragmotica* sp. n.** (Kenya)

**Minor workers** (not known for *Carebara elmenteitae*):

**Table:** 

1	Head weakly subquadratic to subrectangular (CI 90-93), hind femur short (FI 68–69), postpetiole slightly higher than long (LPpI 76–94) and on average about 1.35 times wider than petiole (PpWI 133-137) (Fig. 5)	***Carebara lilith* sp. n.** (Ivory Coast)
–	Head subrectangular (CI 84-88), hind femur moderately short (FI 72-78), postpetiole in profile as long as high or slightly longer (LPpI 100-120) and on average about 1.45 times wider than petiole (PpWI 136-150) (Fig. 7)	***Carebara phragmotica* sp. n.** (Kenya)

**Figure 5.**
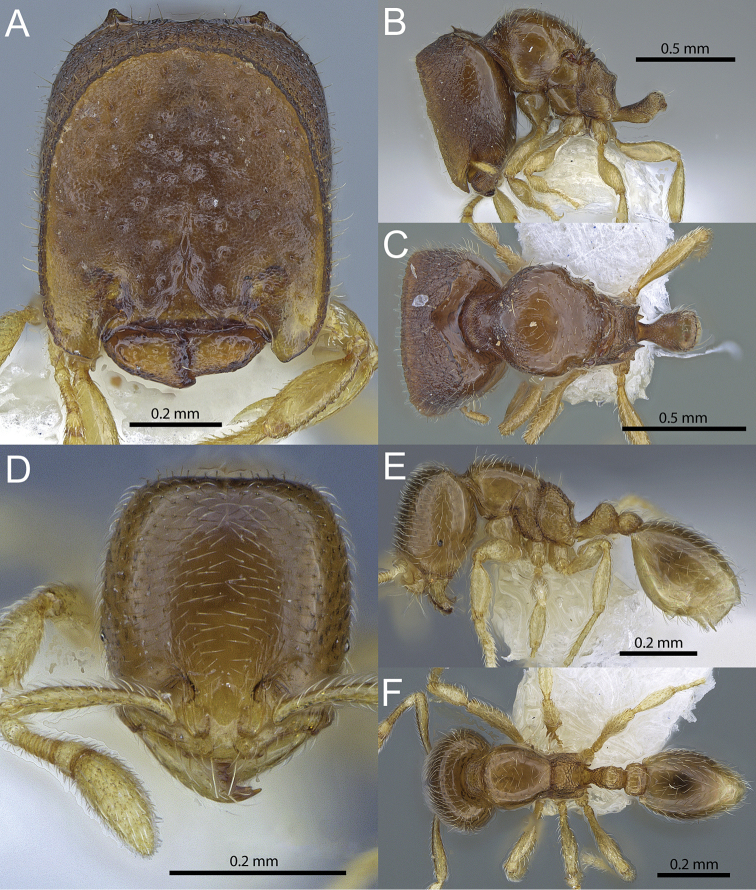
*Carebara lilith* sp. n. Phragmotic worker (holotype: CASENT0709545). **A** head in full-face view **B** body in profile view **C** body in dorsal view. Minor worker (paratype: CASENT0709546) **D** head in full-face view **E** body in profile view **F** body in dorsal view.

**Figure 6.**
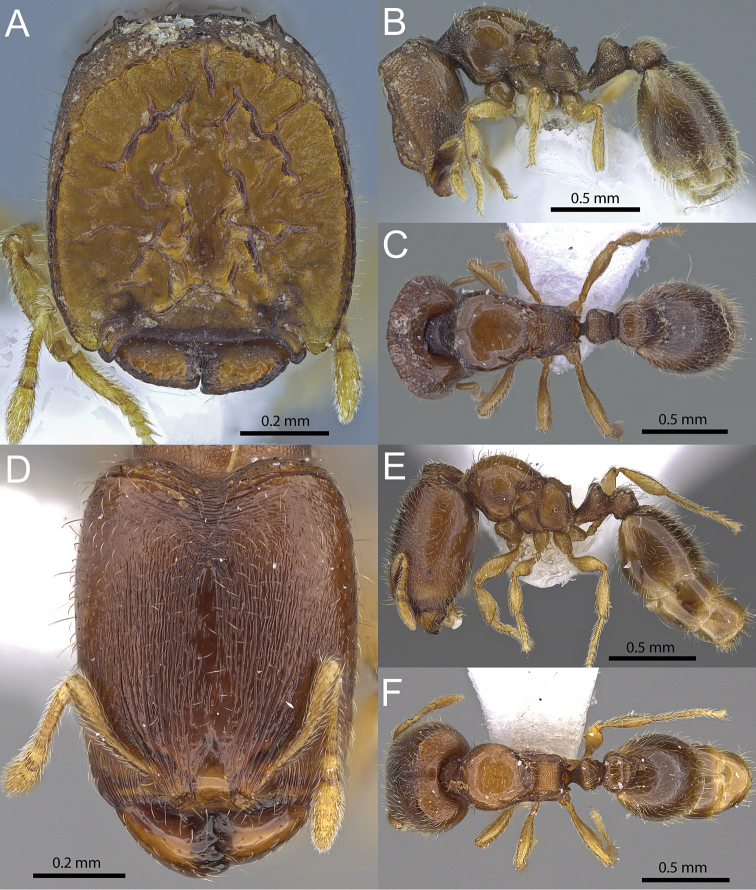
*Carebara phragmotica* sp. n. Phragmotic worker (paratype: CASENT0709550). **A** head in full-face view **B** body in profile view **C** body in dorsal view. Major worker (paratype: CASENT0906158) **D** head in full-face view **E** body in profile view **F** body in dorsal view.

**Figure 7.**
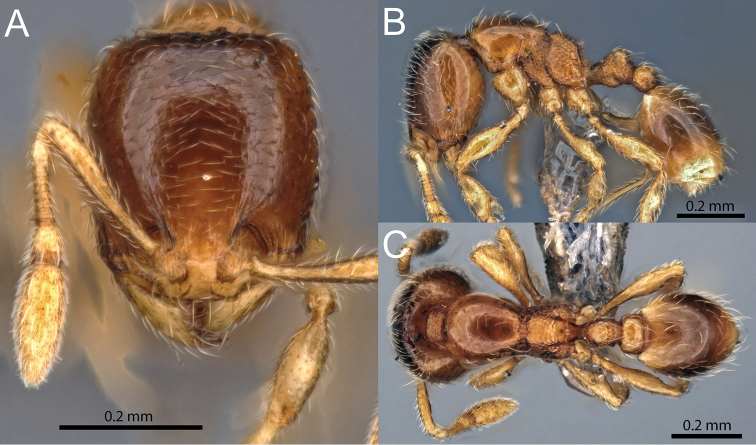
*Carebara phragmotica* sp. n. Minor worker (paratype: CASENT0709554). **A** head in full-face view **B** body in profile view **C** body in dorsal view.

### Species accounts

#### Carebara elmenteitae

Taxon classificationAnimaliaHymenopteraFormicidae

(Patrizi, 1948)

Fig. 3

Solenopsis (Crateropsis) elmenteitae Patrizi, 1948: 176, figs I, II (s.) KENYA. Holotype (IEGG) (Lake) Elmenteita, 20.xii.1945 (*Patrizi*) [not examined]. Combination in *Oligomyrmex*: Ettershank 1966: 123; in *Carebara*: Fernández 2004: 235.

##### Diagnosis.

**Phragmotic worker** (minor and major worker unknown): Head with strongly defined oval cephalic shield, anterolaterally with lobes covering antennae when in repose, mandibles small, clypeus with straight anterior margin and median carina, and anterolateral clypeal lobes either absent or hidden under cephalic shield lobes. Dorsal face of cephalic shield concave, with irregular rugulae or shallow ridges.

##### Distribution.

This species has not been recorded from any locality other than from the type collection near Lake Elmenteita in central Kenya’s Rift Valley.

##### Discussion.

We were not able to examine the holotype specimen from the Patrizi collection in Bologna. Thus, we refrain from a detailed re-description of this species within the present publication and defer to larger-scale future *Carebara* revisions. Therefore, we would like to encourage myrmecologists to collect at or near the type locality in Kenya, which will hopefully lead to findings of additional phragmotic specimens and of the undescribed major and minor worker subcastes. It is also unclear if winged queens exist within this specific clade or if maybe the phragmotic workers are actually ergatoid queens. From the drawings phragmotic workers of *Carebara elmenteitae* can be easily differentiated from those of the new species because of the sculpture inside the cephalic shield: *Carebara elmenteitae* with irregular rugulae or shallow ridges, *Carebara lilith* punctate and with cone-shaped, gland-like structures present, and *Carebara phragmotica* with two subparallel, conspicuously elevated ridges in center of cephalic shield (see also *Carebara lilith* and *Carebara phragmotica* diagnoses and discussions).

#### Carebara lilith

Taxon classificationAnimaliaHymenopteraFormicidae

Fischer, Azorsa & Hita Garcia sp. n.

http://zoobank.org/F797ED46-6335-4C4B-AD19-82CD354BF6CA

Figs 1C, D , 5

##### Holotype.

(major worker), IVORY COAST, Grégbeu, 06.8°, -006.717°, 06.x.1980 (*V. Mahnert & J.-L. Perret*) (CASENT0709545), (MHNG). **Paratypes**: 2 minor workers (CASENT0709546, CASENT0709547), same data as holotype (MHNG).

##### Diagnosis.

**Phragmotic worker**: Anterolateral lobes of clypeus small, shorter than and ending well before anterior margin of lateral shield lobes, sculpture on cephalic shield simple, punctate and with cone-shaped, gland-like structures present. **Major worker**: unknown. **Minor worker**: Head weakly subquadrate to subrectangular (CI 90-93), hind femur short (FI 68-69), postpetiole slightly higher than long (LPpI 76-94) and on average about 1.35 times wider than petiole (PpWI 133-137).

##### Description of phragmotic major worker.

Measurements (n=1): HW 0.65, HL 0.75, SL 0.18, MDL 0.23, EL 0.01, WL 0.73, PNW 0.47, PTL 0.25, PPL -/-, PTH 0.17, PPH -/-, PTW 0.17, PPW -/-, PSL 0.06, MFL 0.36, MTL 0.29, CI 86, SI 28, MDI 35, EI 1, FI 56, PSLI 9.

Head in full-face view modified, phragmotic with a distinct, concave and oval cephalic shield with two forward-extending, semi-transparent anterolateral lobes, inside of cephalic shield in oblique frontolateral view deeply concave and with a sharply raised margin. Head shape in full-face view subrectangular, longer than wide (CI 86), posterior of cephalic shield with rounded posterolateral corners and small horns lateral of shallowly V-shaped posterior emargination. Mandibles reduced and compact (MDI 35). Anterior margin of clypeus straight, widely emarginate and with short anterior lobes lateral of mandibles, which are considerably shorter than and ending before anterior margin of anterolateral cephalic shield lobes. In profile, head anteriorly straight at the truncated cephalic shield margin, short antennal scrobe present ventrally, shielding scrobe and funiculus. Antennae ten-segmented, short, with reduced scape (SI 28), apical funicular segment about as long as the remaining segments combined. Eyes strongly reduced, consisting of one small ommatidium (EI 1), situated at the posterior end of scrobe.

In profile view, promesonotum high and convex, posteriorly roundly sloping towards a very short, partly fused scutellum, both together in dorsal view remotely resembling a diamond-shaped shield. Promesonotal suture absent or inconspicuous, posterior of scutellum a similar-sized, isolated, metanotum present. Propodeal dorsum in profile moderately short, weakly concave towards the short-triangular posterior teeth, posterior declivity almost vertical, with very narrow lamella, propodeal lobe well developed. Propodeal lobes weakly triangular. Propodeal spiracle circular, situated centrally at lateropropodeum.

Petiole in profile with long peduncle, ventrally weakly convex, posterior of small anterior, tooth-like subpetiolar process, the node weakly nodiform or very broadly wedge-shaped, dorsally rounded, anteriorly and posteriorly very weakly concave, in anterodorsal view very weakly convex, almost transverse. Holotype with postpetiole and gaster missing.

Mandibles, clypeus and most of the face finely shagreened, the interior of cephalic shield with many small, cone-shaped, gland-like structures present, posterior portion of clypeus with a short longitudinal carina. Sides of head, lateral to cephalic shield, with weakly reticulate-punctate sculpture, posterior of cephalic shield, towards posterior head margin, longitudinal, weakly reticulate, rugulae present, posterior head margin, between horns, weakly carinate. Ventral side of head smooth and shiny. Promesonotum, anepisternum, katepisternum, propodeal declivity and dorsum of petiole node mostly smooth and shiny; punctures present only at anterolateral promesonotum, at sides and dorsum of propodeum and remainder of petiole, lateropropodeum, below spiracle and near its base, with few longitudinal rugulae.

Lateral and posterior portions of head mostly with short and relatively stout, erect-suberect hairs, no hairs on cephalic shield visible. Mesosoma and petiole node dorsum with abundant, fine, relatively short and mostly decumbent pilosity, plus some longer, subdecumbent to suberect hairs on mesosoma and petiole. Scape and tibia pilosity short, appressed to decumbent. Color light reddish brown, antennae and legs lighter colored.

##### Description of minor workers.

Measurements (n=2): HW 0.29–0.30, HL 0.32–0.33, SL 0.21–0.22, MDL 0.18–0.19, EL 0.02, WL 0.32, PNW 0.19, PTL 0.10–0.13, PPL 0.06, PTH 0.09–0.10, PPH 0.06–0.08, PTW 0.07, PPW 0.09–0.10, PSL 0.04–0.05, MFL 0.20, MTL 0.16–0.17, CI 90–93, SI 73–73, MDI 62–63, EI 5, FI 68–69, PSLI 13–15, LPpI 76–94, DPpI 150–163, PpWI 133–137, PpLI 47–62, PpHI 71–81.

Head longer than wide (CI 90–93), in full–face view weakly subquadrate to subrectangular, with convex sides, posterior head margin straight or very weakly concave medially. Clypeus faintly bicarinate, anterior margin medially very weakly convex or almost transverse. Frontal carinae inconspicuous. Antennae with ten segments, scapes ending before posterior head margin (SI 72–73). Eyes present, consisting of one ommatidium and situated anterior of cephalic midline (EI 5).

In profile view, promesonotum convex, metanotal groove impressed. Propodeum in profile higher than long, weakly convex and declining towards short, acute, weakly triangular posterior teeth, posterior declivity oblique with a narrow lamella and well developed, triangular propodeal lobes. Propodeal spiracle circular, situated just below posterior teeth and very close to posterolateral border of propodeum.

In profile, petiole with moderately short peduncle, ventrally with convex bulge and acute anterior tooth, dorsal face of petiole node more or less convex to weakly wedge-shaped. Postpetiole about as long as high, distinctly lower than petiole (PpHI 71–81), convex dorsally, weakly convex ventrally. In dorsal view petiole node slightly wider than long, postpetiole on average 1.3 times wider than petiole (PpWI 133–137) with sides tapering anteriorly.

Mandibles and clypeus smooth and shiny. Face smooth and shiny, near antennal insertion with few weak, concentric rugulae. Promesonotum, postpetiole dorsum and gaster smooth and shiny, metapleuron, propodeum and petiole with large, partly effaced areolae, propodeal declivity largely smooth and shiny.

Whole body with abundant, relatively short, decumbent pilosity. Clypeus and mesosoma with few longer, suberect hairs present. Scapes and tibiae with short, decumbent pilosity. Color light brown with yellowish antennae and legs.

##### Distribution.

So far, this species is only known from the type locality, although it’s most likely present in unsorted and/or unidentified material in other collections with African ants, possibly collected without phragmotic workers.

##### Discussion.

No ecological or collection data exist for this species. Without the phragmotic major worker, *Carebara lilith* can easily be confused with similar *Carebara* species from the former genus *Oligomyrmex*, as for example *Carebara thoracica*, from which it can be distinguished by possessing ten instead of nine antennal segments. Phragmotic workers of *Carebara lilith* are differentiated from those of *Carebara phragmotica* and *Carebara elmenteitae* by the character combination given in the diagnosis. Morphological differences between minor workers of *Carebara lilith* and *Carebara phragmotica* are not very significant and may decrease even more with larger sample sizes. Especially for the former species, more material is needed for a better resolution of intra- and interspecific variability. Phragmotic workers may be necessary for definitive identifications, but it seems likely that the three species do not co-occur biogeographically.

##### Etymology.

This species is named after the Hebrew name Lilith, a female demon in Jewish mythology. The name is a noun in apposition and thus invariant.

#### Carebara phragmotica

Taxon classificationAnimaliaHymenopteraFormicidae

Fischer, Azorsa & Hita Garcia sp. n.

http://zoobank.org/F36AF74C-CE29-4B5B-9338-DF39C10006CB

Figs 1A, B , 6 , 7

##### Holotype.

phragmotic worker, KENYA, Kakamega Forest, Colobus trail, 000.3551389°, 34.8583611°, 1650m, rainforest, leaf litter, 14.vi.2007 (*M. Peters*) (ZFMK: CASENT0709551).

##### Paratypes.

3 major workers (same data as holotype) (CASENT0906158, CASENT0709548, CASENT0709549); 2 phragmotic workers, Kakamega Forest, Kaimosi fragment, 00.128°, 034.84°, 1600m, rainforest, leaf litter, 04.viii.2008 (*G. Fischer*) (CASENT0709550, CASENT0738556); 1 phragmotic worker, Kakamega Forest, Malawa fragment, 00.4617889°, 034.8587333°, 1650m, rainforest, leaf litter, viii.2007 (*F. Hita Garcia*) (CASENT0277301); 1 phragmotic worker, Kakamega Forest, Yala, 0.202°, 34.868°, 1650m, rainforest, leaf litter, v.2008 (*M. Peters*) (CASENT0738559); 2 major workers, Kakamega Forest, Malawa fragment, 00.4543611°, 034.8635556°, rainforest, leaf litter, 01.ix.2005 (*G. Fischer*) (CASENT0709552, CASENT0709553); 5 minor workers, 1 phragmotic worker, Kakamega Forest, Kisere fragment, 0.385278°, 34.892417°, 1650m, rainforest, leaf litter, 25.xi.2005 (*G. Fischer*) (CASENT0709554, CASENT0709555, CASENT0709556, CASENT0709557, CASENT0709558); 2 minor workers, Kakamega Forest, Salazar, 00.3266667°, 034.8707222°, 1650 m, rainforest, leaf litter, 09.iii.2009 (M. Peters) (CASENT0709559, CASENT0217819); 2 minor workers, Kakamega Forest, Isecheno, 00.235°, 34.869°, 1650m, rainforest, leaf litter, 28.viii.2007 (*F. Hita Garcia*) (CASENT0709560, CASENT0709561); 2 major, 2 minor workers, Kakamega Forest, Kisere fragment (CASENT0709594, CASENT0709595, CASENT0709596, CASENT0709597).

##### Diagnosis.

**Phragmotic worker**: Cephalic shield with two subparallel, conspicuously elevated ridges in its center, in profile distinctly elevated above the rim of the shield. Anterolateral lobes of cephalic shield shorter than and ending before anterior border of clypeal lobes. **Major worker**: Frons and anterior sides of head with abundant, narrow longitudinal rugulae, near posterior head margin a few irregular, weakly defined, transverse rugulae present. **Minor worker**: Head subrectangular (CI 84-88), hind femur moderately short (FI 72-78), postpetiole as long as high or longer (LPpI 100-120) and on average about 1.45 times wider than petiole (PpWI 136-150).

##### Description of phragmotic major workers.

Measurements (n=3): HW 0.68–0.71 (0.70), HL 0.76–0.79 (0.78), SL 0.15–0.23 (0.21), MDL 0.14–0.20 (0.17), EL 0.02, WL 0.78–0.79 (0.79), PNW 0.48–0.50 (0.49), PTL 0.32–0.33 (0.32), PPL 0.15–0.18 (0.17), PTH 0.21–0.22 (0.22), PPH 0.19–0.20 (0.19), PTW 0.20–0.21 (0.21), PPW 0.26–0.27 (0.26), PSL 0.10–0.12 (0.11), MFL 0.39–0.41 (0.40), MTL 0.31–0.32 (0.31), CI 89–90 (89), SI 21–34 (30), MDI 20–28 (24), EI 2, FI 57–59 (58), PSLI 14–17 (15), LPpI 80–96 (88), DPpI 142–170 (156), PpWI 126–129 (127), PpLI 48–55 (52), PpHI 86–90 (88).

Head in full-face view almost as wide as long (CI 89–90), with a phragmotic cephalic shield, outline of shield oval, sharply margined, anterolaterally with short, semi-transparent lobes. Anterior margin of clypeus widely transverse with anteriorly projecting lateral lobes, slightly surpassing the laterally overlapping lobes of the shield.

Cephalic shield with two wavy, sub-parallel, raised ridges centrally, surrounded by several radiating and irregular, shorter canyons and ridges. The inside of the cephalic shield is normally covered with a layer of dirt. Mandibles small, compact (MDI 20-28), and when tightly closed partly hidden under anteriorly projecting clypeus and cephalic shield. Head-shape in profile anteriorly straight along border of cephalic shield, the head appearing like a thick, anteriorly flattened door or plug. Antennal scrobe hidden under anterolateral lobes of cephalic shield. Antennae ten-segmented, short, with reduced scape length as compared to other major workers (SI 21–34), in full-face view largely hidden under cephalic shield. Eyes strongly reduced, consisting of one small ommatidium (EI 2), situated at the posterior end of scrobe.

In profile view, promesonotum high and convex, posteriorly sloping linearly towards a short, separated or anteriorly fused, posteriorly sharply margined scutellum, in dorsal view comparable to a polished convex shield. Promesonotal suture absent or inconspicuous, scutellum small and weakly to not isolated in dorsal view, metanotum present as a small bump extending dorsally between propodeum and scutellum, metanotal groove narrowly impressed. Propodeal dorsum short, anteriorly convex, with a blunt angle halfway towards the short, bluntly triangular to rounded propodeal teeth, posterior declivity oblique, with short and narrow lamella and well developed, lamellate propodeal lobe. Propodeal spiracle circular, situated close to center of lateropropodeum.

Petiole in profile with relatively short peduncle, ventrally straight to weakly convex, with small to reduced anterior tooth, lateroventral margins posteriorly with very thin, elongate lamellae present, the node sub-triangular and dorsally rounded to very broadly wedge-shaped, postpetiole in profile higher than long (LPpI 80–96) and almost as high as petiole (PpHI 86–96), its dorsum convex and with a very short ventral face. In dorsal view, petiole node shape transversely oval, wider than long and posteriorly flattened, postpetiole wider than long, suboval, anterior margin concave and posterior margin almost straight, about 1.3 times wider than petiole (PpWI 126–129).

Mandibles, clypeus and most of the cephalic shield finely shagreened, highest areas on central ridges smooth and shiny. Posterior of cephalic shield, near head margin, shagreening overlain with weakly reticulate rugulae, posterior head margin with weakly raised carina and small horns present at posterolateral corners. Ventral side of head very finely and obliquely striate. Promesonotum, parts of anepisternum and katepisternum, postpetiole dorsum and gaster mostly smooth and shiny, remainder of body punctate.

Lateral and posterior portions of head with very short, erect to suberect hairs, no visible hairs on cephalic shield, mesosoma with relatively sparse, fine, relatively short, decumbent pilosity, and few longer, subdecumbent to suberect fine hairs. Waist segments and gaster covered with very abundant pilosity, apical segments of gaster also with many suberect, long standing hairs. Scape and tibiae pilosity abundant and decumbent. Color light or reddish brown, antennae, legs and parts of gaster, yellow.

##### Description of major workers.

Measurements (n=5): HW 0.59–0.63 (0.61), HL 0.74–0.78 (0.76), SL 0.27–0.30 (0.29), MDL 0.32–0.34 (0.33), EL 0.03, WL 0.64–0.67 (0.65), PNW 0.36–0.39 (0.37), PTL 0.26–0.27 (0.26), PPL 0.14–0.17 (0.15), PTH 0.19–0.20 (0.21), PPH 0.17–0.19 (0.18), PTW 0.18–0.20 (0.19), PPW 0.23–0.25 (0.23), PSL 0.08–0.10 (0.09), MFL 0.34–0.38 (0.36), MTL 0.26–0.29 (0.28), CI 79–82 (80), SI 46–49 (47), MDI 53–55 (54), EI 5, FI 58–62 (59), PSLI 13–15 (14), LPpI 80–92 (87), DPpI 141–160 (152), PpWI 119–133 (126), PpLI 56–61 (58), PpHI 86–96 (90).

Head in full-face view rectangular, about 1.25 times longer than wide (CI 79–82), sides subparallel, posterior margin with transverse carina present on either sice of narrow, evenly concave median emargination, horns small and obtuse, posterolateral corners rounded. Mandibles triangular, about half as long as head width, masticatory margin with five teeth including basal tooth. Frontal carinae absent or inconspicuous. Anterior margin of clypeus concave medially and laterally on either side of median concavity. Frons sometimes with median ocellus present. Antennae with ten segments. Scapes short and when laid back not surpassing cephalic midlength (SI 46–49). Eyes present, consisting of one relatively large ommatidium (EI 5).

In profile, pronotum high and convex, posteriorly declining linearly, propodeum distinctly higher than long, dorsal face obliquely declining, posterior corners either edentate and angulate or with very small triangular teeth, posterior declivity nearly vertical with very shallow lamella, propodeal lobes relatively small. Pronotum in dorsal view strongly rounded and almost circular, pronotal suture on dorsum inconspicuous or present as weak impression, scutellum very small and often fused with pronotum, metanotal groove present, barely or not impressed, propodeal spiracle circular, situated almost at center of lateropropodeum.

Petiole in profile with short peduncle, almost subtriangular in shape, posteroventrally weakly convex, anteriorly with small tooth or subpetiolar process. Petiole node dorsally flat to weakly convex, in dorsal view much wider than long, its posterior end well defined to weakly marginate. Postpetiole in profile higher than long (LPpI 80–92), almost as high as petiole (PpHI 86–96), convex dorsally, and with a small, angulate ventral process. In dorsal view postpetiole wider than long, suboval with rounded sides, on average 1.3 times wider than petiole (PpWI 119–133).

Mandibles smooth and shiny with scattered, short, appressed pilosity and weak, short rugulae laterally near their bases. Head with very fine and densely packed longitudinal striations, near posterior margin replaced by irregular transverse rugulae, with a conspicuously raised and transversely curved carina laterally of median concavity. Mesosoma and petiole mostly weakly punctate, except for smooth and shiny promesonotal dorsum and parts of lateropronotum, with rugosities at junctions of pronotum, anepisternum, katepisternum and metapleuron. Postpetiole dorsally smooth and shiny, its remainder punctate. Gaster smoth and shiny.

Head and body with abundant, moderately long, decumbent to subdecumbent pubescence and with few suberect hairs. Scape and tibia pilosity abundant and decumbent. Color brown to light brown, antennae, legs and parts of gaster, slightly lighter.

##### Description of minor workers.

Measurements (n=5): HW 0.32–0.34 (0.33), HL 0.38–0.39 (0.38), SL 0.23–0.25 (0.24), MDL 0.20–0.28 (0.23), EL 0.02, WL 0.38–0.39 (0.39), PNW 0.21–0.23 (0.22), PTL 0.14–0.16 (0.15), PPL 0.08–0.09 (0.08), PTH 0.11–0.12 (0.12), PPH 0.08–0.09 (0.08), PTW 0.08–0.09 (0.08), PPW 0.11–0.12 (0.11), PSL 0.05, MFL 0.24–0.26 (0.25), MTL 0.19–0.20 (0.19), CI 84–88 (86), SI 70–75 (73), MDI 60–82 (69), EI 4–7 (6), FI 72–78 (74), PSLI 13–15 (14), LPpI 100–120 (105), DPpI 133–150 (140), PpWI 136–150 (145), PpLI 53–61 (56), PpHI 67–74 (70).

Head longer than wide (CI 84–88), in full–face view weakly subrectangular, sides convex, posterior margin nearly straight to faintly convex. Anterior margin of clypeus straight medially, weakly bicarinate, and narrow between antennal insertions. Frontal carinae very weakly developed, ending at or before eye level. Antenna with ten segments, scapes, when laid back, ending well before posterior head margin (SI 70–75). Eyes small (EI 4–7), consisting of one ommatidium, situated slightly anterior to cephalic midline.

In profile, promesonotum convex, metanotal groove impressed, propodeal dorsum weakly convex, shorter than posterior declivity, declining posteriorly towards small, acute to bluntly triangular, lamellate propodeal teeth, declivity of propodeum oblique, with narrow lamella connecting the teeth and relatively large propodeal lobes. Propodeal spiracle circular and situated close to posterior border of propodeum, just below the propodeal teeth.

Petiole in profile view with a short peduncle, with a convex ventral bulge, anteriorly with a short, triangular tooth, petiole node dorsally roundly subtriangular. Postpetiole relatively short and low, as long as high or slightly longer (LpPI 100–120), lower than petiole (PpHI 67–74), dorsum convex. In dorsal view petiole node about as wide as long, postpetiole subrectangular with rounded corners, about 1.45 times wider than petiole (PpWI 136–150).

Mandibles and clypeus smooth and shiny. Face smooth and shiny, near antennal insertion with weak concentric carinae, frontal carinae very short and reduced. Promesonotum, postpetiole dorsum, most of propodeal declivity, and gaster smooth and shiny, metapleuron, propodeum and petiole areolate.

Whole body with abundant, relatively short, decumbent pilosity. Clypeus and body with very few erect to suberect hairs present. Scape pilosity short, decumbent to subdecumbent, tibia pilosity mostly decumbent. Color orange to light brown, with yellowish antennae and legs.

##### Distribution.

This species is known only from Kakamega Forest and its smaller fragments in the north (Malawa and Kisere Forest) and in the south (Kaimosi Forest), in the Western Province of Kenya.

##### Discussion.

*Carebara phragmotica* is different from *Carebara elmenteitae* (Patrizi), which was collected in leaf-litter on the banks of the river Kariandus near Lake Elementeita in the Great Rift Escarpment in Kenya (Patrizi 1948), and that has been described from a single phragmotic worker. Interestingly the specimen was identified as a queen, but according to its’ description and the drawing it very closely resembles the phragmotic major workers of *Carebara phragmotica* sp. n. and *Carebara lilith* sp. n. Despite their high morphological similarity, the phragmotic majors of these three species can be separated from each other by the following head characters: horns at posterior head margin and anterolateral lobes of clypeus lacking or indistinct in *Carebara elmenteitae* versus horns distinct in *Carebara phragmotica* and *Carebara lilith*, and the clypeal lobes anteriorly surpassing the lateral lobes of the relatively small cephalic shield lobes in *Carebara phragmotica* versus the cephalic shield lobes significantly larger and anteriorly surpassing the clypeal lobes in *Carebara lilith*. Finally, the cephalic shield in *Carebara elmenteitae* contains low, irregular rugulae or ridges, versus the two highly raised, subparallel central ridges, that, in profile, are distinctly higher than the rim of the cephalic shield in *Carebara phragmotica*, whereas *Carebara lilith* is characterized by a mostly flat cephalic shield surface without ridges or canyons, instead with punctures and small, cone-shaped structures present.

Minor workers of *Carebara phragmotica* can be differentiated from those of *Carebara lilith* by the characters listed in the diagnosis (see discussion of *Carebara lilith*). Minor and major workers of *Carebara phragmotica* can be distinguished from those of *Carebara thoracica* by their antennal segmentation (ten segments in *phragmotica* versus nine in *thoracica*).

##### Etymology.

This species’ name is derived from the modified head morphology of the phragmotic major workers.

## Supplementary Material

XML Treatment for Carebara elmenteitae

XML Treatment for Carebara lilith

XML Treatment for Carebara phragmotica
